# High sensitivity of one-step real-time reverse transcription quantitative PCR to detect low virus titers in large mosquito pools

**DOI:** 10.1186/s13071-020-04327-4

**Published:** 2020-09-09

**Authors:** Zhaoyang Tang, Hanano Yamada, Carina Kraupa, Sumejja Canic, Núria Busquets, Sandra Talavera, Davy Jiolle, Marc J. B. Vreysen, Jérémy Bouyer, Adly M. M. Abd-Alla

**Affiliations:** 1 Insect Pest Control Laboratory, Joint FAO/IAEA Division of Nuclear Techniques in Food and Agriculture, Vienna International Centre, P.O. Box 100, 1400 Vienna, Austria; 2 Key Laboratory of Vector Biology and Pathogen Control of Zhejiang Province, College of Life Sciences, Huzhou University, Huzhou, 313000 China; 3 IRTA, Centre de Recerca en Sanitat Animal (CReSA, IRTA-UAB), Campus de la Universitat Autònoma de Barcelona, 08193 Bellaterra, Spain; 4 UMR MIVEGEC (IRD 224-CNRS 5290-UM), Maladies Infectieuses et Vecteurs: Ecologie Génétique, Evolution et Contrôle, Institut de Recherche pour le Développement (IRD), Montpellier, France

**Keywords:** Flavivirus, Arbovirus, Chikungunya virus (CHIKV), Usutu virus (USUV), West Nile virus (WNV), Zika virus (ZIKV), Pool size, RT-qPCR

## Abstract

**Background:**

Mosquitoes are the deadliest animals in the world. Their ability to carry and spread diseases to humans causes millions of deaths every year. Due to the lack of efficient vaccines, the control of mosquito-borne diseases primarily relies on the management of the vector. Traditional control methods are insufficient to control mosquito populations. The sterile insect technique (SIT) is an additional control method that can be combined with other control tactics to suppress specific mosquito populations. The SIT requires the mass-rearing and release of sterile males with the aim to induce sterility in the wild female population. Samples collected from the environment for laboratory colonization, as well as the released males, should be free from mosquito-borne viruses (MBV). Therefore, efficient detection methods with defined detection limits for MBV are required. Although a one-step reverse transcriptase quantitative polymerase chain reaction (RT-qPCR) method was developed to detect arboviruses in human and mosquito samples, its detection limit in mosquito samples has yet to be defined.

**Methods:**

We evaluated the detection sensitivity of one step RT-qPCR for targeted arboviruses in large mosquito pools, using pools of non-infected mosquitoes of various sizes (165, 320 and 1600 mosquitoes) containing one infected mosquito body with defined virus titers of chikungunya virus (CHIKV), usutu virus (USUV), West Nile virus (WNV) and Zika virus (ZIKV).

**Results:**

CHIK, USUV, ZIKV, and WNV virus were detected in all tested pools using the RT-qPCR assay. Moreover, in the largest mosquito pools (1600 mosquitoes), RT-qPCR was able to detect the targeted viruses using different total RNA quantities (10, 1 and 0.1 ng per reaction) as a template. Correlating the virus titer with the total RNA quantity allowed the prediction of the maximum number of mosquitoes per pool in which the RT-qPCR can theoretically detect the virus infection.

**Conclusions:**

Mosquito-borne viruses can be reliably detected by RT-qPCR assay in pools of mosquitoes exceeding 1000 specimens. This will represent an important step to expand pathogen-free colonies for mass-rearing sterile males for programmes that have a SIT component by reducing the time and the manpower needed to conduct this quality control process.
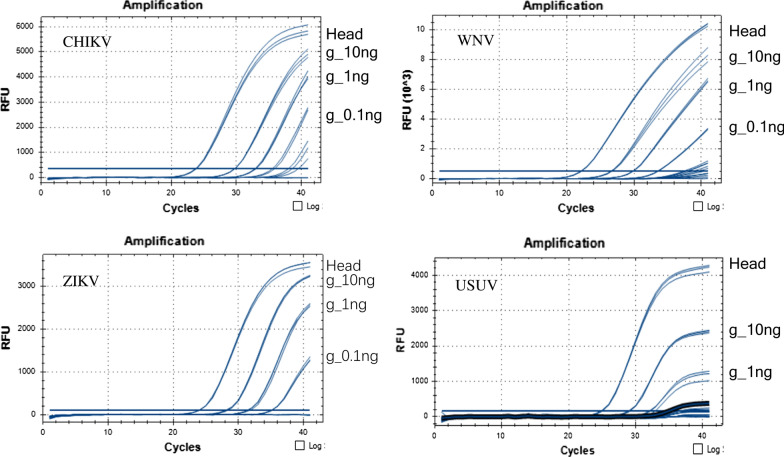

## Background

Mosquitoes are a group of vectors that transmit an array of human viruses. Most of mosquito-borne viruses (MBVs) belong to the families *Flaviviridae*, *Togaviridae* and *Bunyaviridae* and can cause severe human diseases including haemorrhagic fever, biphasic fever, encephalitis, and meningitis [1]. Hundreds of millions of infections are caused by MBVs annually [2]. Dengue virus (DENV), chikungunya virus (CHIKV), West Nile virus (WNV) and Zika virus (ZIKV) are the most prevalent arboviruses in the world [3]. Emerging, and re-emerging arboviral infections have caused substantial public concerns in recent years [4]. For example, DENVs are estimated to infect around 400 million people per year, and over half of the world’s population is at risk of the disease [5]. Chikungunya virus emerged from Africa in the mid 2000s, spreading first across India and Asia and then into the Americas in 2013 [6]. West Nile virus was first isolated from a human in 1937. Since then, its distribution has expanded to all continents except the arctic regions [7]. Zika virus outbreaks occurred in the South Pacific in 2013 and in the Americas in 2015 [6]. Usutu virus (USUV) is an emerging mosquito-borne flavivirus belonging to the *Flaviviridae* family [8], and is closely related to Murry Valley encephalitis virus and WNV [8]. USUV virus has been found to co-circulate with WNV in Europe and one asymptomatic blood donor in Austria was found to be potentially co-infected with both viruses [9, 10].

No effective antiviral drugs or vaccines are currently available for most of the MBVs except the yellow fever virus, but they can be prevented by avoiding mosquito bites [11], and hence, effective mosquito-control methods are urgently needed. Traditional mosquito control methods include source reduction by removing the breeding sites and the use of chemical insecticides including the application of insecticide-treated nets (ITNs) and indoor residual spraying (IRS). Although these strategies, in addition to various biological control tactics (such as the use of larvivorous fish in larval breeding sites) and personal protection measures have been effective in the past, they have shown limited sustainability, and they have not been able to prevent the proliferation and spread of mosquito populations and their associated MBVs. For the sterile insect technique (SIT), male insects are irradiated with ionising radiation that creates dominant lethal mutations in the cells, rendering them infertile. The mating of sterile males with wild females will induce sterility in the wild female target insect population, as these fail to produce viable offspring. This method has successfully suppressed or locally eradicated populations of selected insect pests [12, 13]. It represents an additional control tactic that can be combined with other suppression tools for sustainable mosquito population management to protect human health and the environment. Combining the incompatible insect technique (IIT) with the SIT enabled suppression of field populations of *Aedes albopictus*, the world’s most invasive mosquito species, in two isolated villages in China [14]. Millions of factory-reared adult males were released in the field to compete for, and mate with wild females, resulting in non-viable eggs. Inundative, sequential releases of competitive sterile males over many generations resulted in a significant reduction of the wild population.

The SIT requires the establishment of a mother colony from wild-collected mosquitoes before up-scaling and ultimately mass-rearing to produce sterile males for release. The collection of wild mosquitoes for this purpose holds the risk of initiating the mother colony with individuals that are infected with viruses, as MBVs are widespread in most regions. For this reason, a sensitive detection method is crucial to screen wild-collected mosquitoes and ensure that samples are virus-free before establishing the mother colony in the insectary. Furthermore, periodic screening of the colonies and mass-reared material is important to ensure the absence of any accidental contamination of the colonies by any MBVs. Such screening as part of quality control in mass-rearing facilities is not only essential to ensure adequate biosafety for insectary staff, but also to ensure the general public that any females accidentally released together with the sterile males released during SIT programmes are pathogen-free.

RT-qPCR is a highly sensitive and specific assay for the identification and detection of several RNA viruses such as CHIKV [15], WNV [16], USUV [17] and ZIKV [18] and the detection limits for some of these viruses have been determined. The dengue virus is a single positive-stranded RNA virus in the genus *Flavivirus* and includes four DENV serotypes; DEN-1, DENV-2, DENV-3 and DENV-4. A real-time RT quantitative PCR has been developed to detect viral RNA of each DEN serotype. In single reactions and in fourplex reactions (containing four primer-probe sets in a single reaction mixture), standard dilutions of virus equivalent to 0.002 plaque forming unit (PFU) of DENV-2, DENV-3, and DENV-4 viruses were detected, and the limit of detection of DENV-1 virus was 0.5 equivalent PFU [19].

Previous studies of ZIKV detection indicate that viral concentrations vary between sample matrices, such as blood, urine, or saliva. In the case of urine and saliva, the lowest viral RNA detected was reported to be 10^2^ copies/ml in urine and 40 copies/ml in saliva with their highest range being 2.68 × 10^3^ copies/ml and 7.44 × 10^4^ copies/ml, respectively [20].

Lanciotti et al. [16] reported a rapid TaqMan assay for the detection of WNV in a variety of human clinical specimens and field-collected mosquitoes. The RT-qPCR was specific for WNV and detected 0.1 PFU with greater sensitivity than the traditional RT-qPCR method. Nikolay et al. [17] presented a quantitative real-time RT-qPCR assay for USUV based on conserved regions from Europe and Africa. The assay provides high analytical specificity for USUV and 60 copies/reaction for the RNA standard.

Although several molecular tests have been published for detecting MBVs, few have reported the use of these tests for detecting MBVs in mosquito samples. Sutherland et al. [21] conducted a laboratory evaluation of the ability of commercial antigen-capture assays, the rapid analyte measurement platform (RAMPH) and the VecTestH wicking assay, as well as real time reverse transcriptase polymerase chain reaction (RT-qPCR, TaqMan) and Vero cell plaque assay to detect WNV in large mosquito pools. Real-time PCR (TaqMan) was the most sensitive, detecting WNV ribonucleic acid (RNA) in 100% of the samples containing a single infected mosquito in pool sizes of up to 500 mosquitoes. Mosquito body tissues minimally impacted the ability of RT-qPCR to detect WNV in a pool size of 500, with a sensitivity of 0.6 log_10_ PFU/ml.

This study aimed to evaluate the one-step real-time reverse transcription quantitative PCR method for the screening of mosquito pools and to establish detection limits of different MBVs for the purpose of effective periodic screening of mosquito colonies and released insects in the context of applying the SIT against disease-transmitting mosquito populations. The purpose of this article was to determine the extent that pool size could be increased while still maintaining the ability to detect one infected individual.

## Methods

### Mosquito species

The non-infected *Aedes albopictus* strains used in the present study were maintained in the bio-secure insectary of the Insect Pest Control Laboratory (IPCL), Joint FAO/IAEA Division of Nuclear Techniques in Food and Agriculture, Seibersdorf, Austria and reared following the FAO/IAEA guidelines [22]. In brief, mosquito strains were kept under standard laboratory conditions at a temperature of 27 ± 1 °C, 60 ± 10% relative humidity, and a photoperiod of 12:12 (L:D) h including dusk (1 h) and dawn (1 h) transitional periods [22, 23]. Adults were kept in standard 30 × 30 × 30 cm Bugdorm cages (Megaview Science Education Services Co. Ltd., Taichung, Taiwan) in an insectary deprived of natural light and continuously supplied with 10% wt:vol sucrose solution. Before total RNA extraction, adults were starved for 12 h to empty stomach content and stored at − 80 °C.

### Virus-infected mosquito samples

The project ‘Research infrastructures for the control of vector-borne diseases’ (lnfravec2, https://infravec2.eu/) provided the BORA strain of *Ae. aegypti*, infected with CHIKV and ZIKVs, and the Gavà strain of *Culex pipiens*, infected with WNV and USUV. In brief, 5–7-day old females of the *Ae. aegypti* BORA strain were infected with CHIKV and ZIKV by feeding them on an infectious blood meal with virus titers of 1.5 × 10^7^ and 1.02 × 10^7^ plaque-forming units (PFU)/ml respectively. The virus titration was performed by the plaque assay for CHIKV and ZIKV and expressed in PFU/ml as previously described [24, 25]. A Hemotek^®^ system (Discovery Workshops, Accrington, UK) was used for feeding the adult females and engorged females were fed with 10% sucrose in a chamber incubated at 28 °C and 80% humidity for 14 days in the bio-secure insectary of the Maladies Infectieuses et Vecteurs: Ecologie, Génétique, Evolution et Contrôle, Institut de Recherche pour le Développement (IRD), Montpellier, France. In addition, *Cx. pipiens* females inoculated with WNV and USUV were prepared at the Centre de Recerca en Sanitat Animal (Campus of Autonomous University of Barcelona) Barcelona, Spain. *Culex pipiens* females were inoculated intrathoracically with 1–2 µl per adult using a stock of WNV (7.52 log_10_ TCID_50_/ml) and USUV (6.88 log_10_ TCID_50_/ml). Virus titers were determined by a standard limiting dilution assay [26] using monolayers of cells employed for virus propagation. Viral titers were expressed in 50% tissue culture infectious dose (TCID_50_) per ml. Injected females were kept at 28 °C and 80% humidity for 7 days. Bodies and heads were separated in individual tubes and homogenized in 500 µl of TRIzol (Invitrogen, Carlsbad, California, USA) and kept at − 80 °C. Infected heads and bodies were delivered to the IPCL for RNA extraction and further analysis. The level of infection of infected mosquito bodies was estimated by evaluating the virus infection titer in the corresponding head using quantitative RT-qPCR. Only bodies of which the corresponding head showed high virus titer were considered infected and were used in spiking the non-infected mosquito samples.

### Spiking non-infected mosquito samples with virus-infected mosquitos

To determine the ability of the quantitative RT-qPCR assays to detect MBVs, a single *Ae. aegypti* infected with CHIKV or ZIKV and *Cx. pipiens* infected with WNV or USUV, was homogenized within large pools of uninfected adult *Ae. albopictus* mosquitoes, i.e. *n* = 165 (100 mg), *n* = 320 (200 mg) and *n* = 1600 (1000 mg) and total RNA extracted. A pool consisting of only uninfected mosquitoes was included as a negative control. The negative control, and the 320 and 1600 mosquito pools were replicated twice.

### Total RNA extraction

Total RNA was extracted from mosquitoes using TRIzol Reagent (Invitrogen, Carlsbad, California, USA) according to the supplier’s instructions. Based on the size of the pool, adult mosquitoes were homogenized either in microtubes with a sterile pestle or with a sterilised mortar and pestle after adding liquid nitrogen. After grinding and homogenizing mosquito adult pools using liquid nitrogen, TRIzol reagent containing one single infected mosquito was added to the uninfected mosquito pool and the RNA extraction was carried out. For the negative control pool, TRIzol reagent was added after grinding with liquid nitrogen. The total RNA pellet was resuspended in 100, 200 and 1000 µl of RNase-free water for the pools composed of 165, 320 and 1600 mosquitoes, respectively. The quantity and quality of RNA samples were determined using a Synergy™ H1 microplate reader (BioTek, Winooski, Vermont, USA). The RNA samples were serially diluted in ten-fold steps from 10 to 0.0001 ng/µl for a concentration that will consistently give the same amount per well in the RT-qPCR and were stored at − 80 °C.

### Evaluation of primers and probes specificity

To investigate the possibility of using multiple primers and probes sets to detect several viruses in the same reaction, we tested the specificity of each primer and probe set to detect other MBVs. The total RNA extracted from uninfected mosquitoes was tested to ensure that there were no false positives caused by cross-reactions with the host-species. This step also determined any background signal generated by primer cross-reactivity with mosquito-derived RNA.

### Generation of standard curves for the RT-qPCR

To obtain a stable positive control for the detection of MBVs, the RNA sequences containing and flanking the sequence regions of the virus specific primers and probes of CHIKV, ZIKV, WNV and USUV were amplified using the primers listed in Table 1 and cloned into pGEM-T vector (Promega, Madison, Wisconsin, USA). The primers in Table 1 were selected using the primer3 software with the default setting (http://bioinfo.ut.ee/primer3-0.4.0/) and the sequence of CHIKV (GenBank: NC004162), USUV (GenBank: AY453412), ZIKV (GenBank: AY632535) and WNV (GenBank: DQ211652). The SuperScript^®^ III First-Strand Synthesis System for RT-qPCR (Invitrogen) was used to synthesize first-strand cDNA from purified poly(A)+ or total RNA following the manufacturer’s instructions. The targeted sequences were amplified by Taq PCR Master Mix (Qiagen) with the following PCR conditions: 5 min at 94 °C; 35 cycles of 30 s at 94 °C, 30 s at 58 °C and 1 min at 72 °C; and 10 min at 72 °C. The PCR product was purified using the High Pure PCR Clean-up Micro Kit (Roche, Basel, Switzerland) and ligated to the pGEM-T vector (Promega, Madison, Wisconsin, USA), following the supplier’s instructions. The recombinant plasmids were transformed into DH5α competent bacteria (Invitrogen, Carlsbad, California, USA) following the supplier’s instructions. The recombinant plasmids and the inserted sequences were confirmed by Sanger sequencing (Eurofins Genomics, Ebersberg, Germany) with the universal vector primers M13F_uni (-21) (5′-TGT AAA ACG ACG GCC AGT-3′) and M13R_rev (-29) (5′-CAG GAA ACA GCT ATG ACC-3′). Recombinant plasmids were amplified, and the quantity and quality were determined using a Synergy™ H1 microplate reader (BioTek, Winooski, Vermont, USA). The DNA copy number was estimated using NEBioCalculator (https://nebiocalculator.neb.com/#!/dsdnaamt), then 7 concentrations with known DNA copy numbers/µl were prepared by serial dilutions and used to estimate the virus copy number in infected and non-infected mosquito samples. Sterile, nuclease-free water was used as a no template control (NTC), then tested in triplicates.

**Table 1:** Primers for viral cloning in pGEM-T vector

Oligo name	Sequence (5′–3′)	Size (bp)	Genome position	GenBank ID
Usu_9814F	GTGCCTTTCTGCTCAAACCA	585	9814–9833	AY453412
Usu_10398R	CAAAACCCTGTCCTCCTGGAC		10,378–10,398	
ZIKV_816F	CAAGAGAATACACRAAGCACTTGA	539	816–839	AY632535
ZIKV_1365R	ATGCTCTTCCCGGTCATYTTCT		1344–1365	
CHIKV_645F	GTGCCTACCCCTCATACTCG	553	645–664	NC_004162
CHIKV_1198R	CCGTTGCGTTCTGCCGTTA		1180–1198	
WNV_10533F	AAGTTGAGTAGACGGTGCTG	340	10,533–10,552	DQ211652
WNV_10873R	TTCCCCTGACCTACAGCTTC		10,854–10,873	

### One-step real-time RT-qPCR

Mosquito-borne virus-specific primers and TaqMan probes previously reported to detect each specific virus were synthesized by Eurofins Genomics with 5-FAM, HEX as the reporter dye for the probe. The details of the primers and probes sequences and their characteristics are shown in Table 2. The real-time RT-qPCR assay was performed using a CFX96 Real-Time System cycler (Bio-Rad, Hercules, California, USA) and the Quantitect Probe RT-qPCR kit (Qiagen). Reactions were performed in a 20 µl volume mixture containing 1 µl of RNA template, 10 µl of 2× QuantiTect Probe RT-qPCR Master Mix, 8 µM of forward primer, 8 µM of reverse primer, 2.5 µM of probe, 0.25 µl of QuantiTect RT Mix and 5.95 µl RNase-free water. The amplification of the cDNA and the quantification of the viral copy number was done in one step using the following protocol: a single cycle of reverse transcript for 15 min at 50 °C, 15 min at 95 °C and 40 cycles of 15 s at 95 °C and 1 min at 60 °C. The real-time data were analysed using the CFX manager software provided by Bio-Rad. Negative and positive controls were included in all PCR reactions performed. A sample was determined empirically to be positive if the Cq value was 36, based on background cross-reactivity of the primers and probes in non-template control reactions. Positive results were determined according to the amplification cycle at which the relative fluorescence unit (RFU) was detected below the quantification cycle (Cq). Baseline thresholds for the two fluorophores were determined with the CFX manager software in a series of reactions using the virus standard dilutions and then set for subsequent runs as auto calculated [27].

**Table 2:** Nucleotide sequences of primers and probes used in RT-qPCR assays

Oligo name	Sequence (5′–3′)	Modification		Genome position	GenBank ID	References
		5’	3’			
Usu_F	CAAAGCTGGACAGACATCCCTTAC			10,189–10,212	AY453412	[17]
Usu_R	CGTAGATGTTTTCAGCCCACGT			10,270–10,291		
Usu_Probe	AAGACATATGGTGTGGAAGCCTGATAGGCA	6FAM	TMR	10,226–10,255		
ZIKV_F	CCGCTGCCCAACACAAG			1191–1208	AY632535	[18]
ZIKV_R	CCACTAACGTTCTTTTGCAGACAT			1245–1268		
ZIKV_Probe	AGCCTACCTTGACAAGCAGTCAGACACTCAA	5′-FAM	3′-TAMRA	1213–1243		
CHIKV874	AAAGGGCAAACTCAGCTTCAC			874–894	NC_004162	[15]
CHIKV961	GCCTGGGCTCATCGTTATTC			942–961		
CHIKV899-probe	CGCTGTGATACAGTGGTTTCGTGTG	5′-FAM	3′-BHQ1	899–923		
WN3′NC-F	CAGACCACGCTACGGCG			10,668–10,684	DQ211652	[16]
WN3′NC-R	CTAGGGCCGCGTGGG			10,770–10,756		
WN3′NC-probe	TCTGCGGAGAGTGCAGTCTGCGAT	5′-FAM	TAMRA	10,691–10,714		

## Results

### Calibration curve

**Fig. 1:**
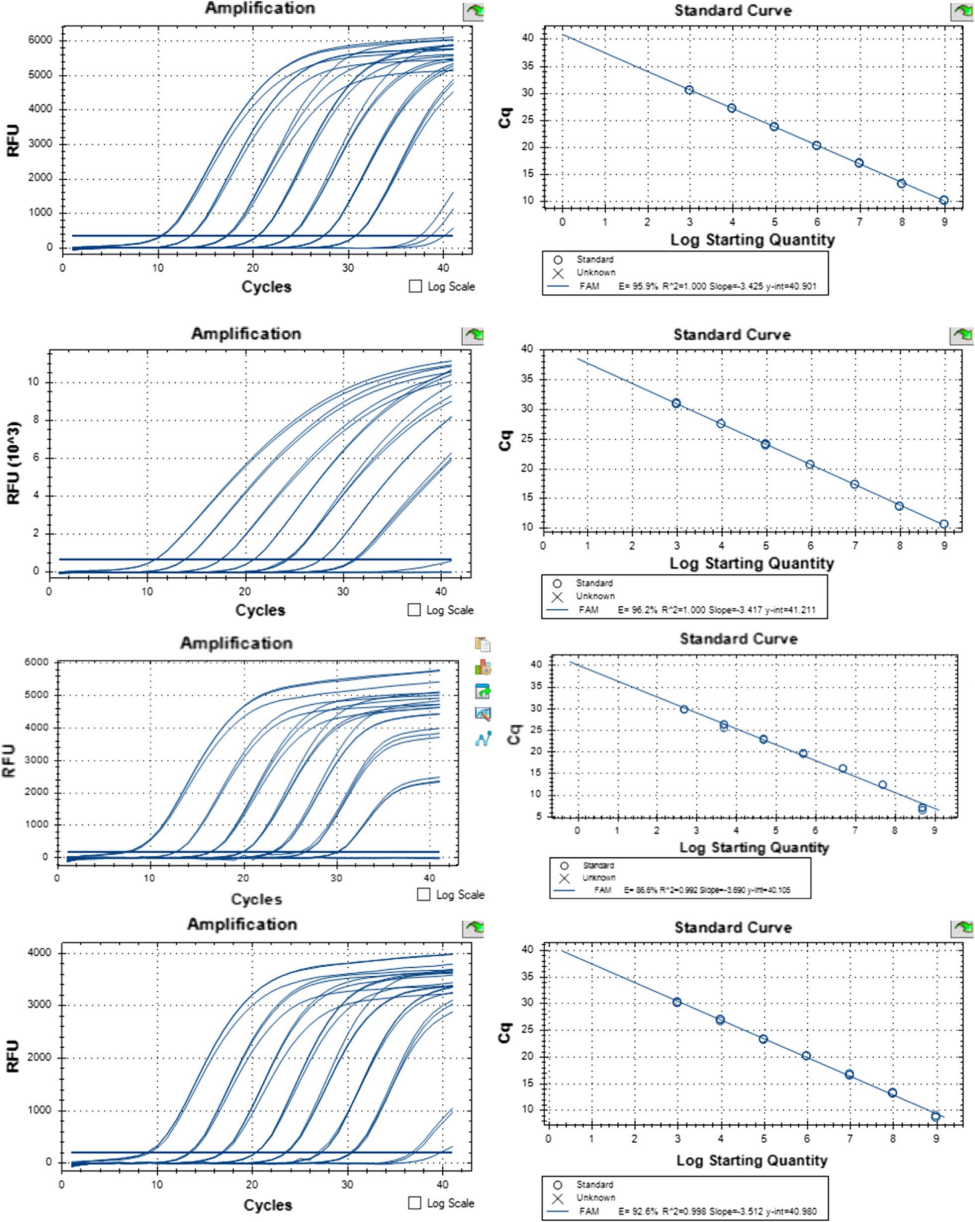
Amplification and standard curves of serial dilution of plasmid containing the sequence targeted by the primers and probes for the RT-qPCR for CHIKV, WNV, USUV and ZIKV detection. The correlation between the relative florescent unit (RFU) and the quantification cycle (Cq) on the left and between the virus log 10 copy number and the Cq on the right. Measurements were taken in triplicates. The regression equations and correlation coefficients (*R*) are given for each plot

The sequence analysis of the recombinant plasmids confirmed the presence of the targeted sequence of CHIKV, ZIKV, USUV and WNV which correctly matched with the virus sequence available in the sequence database. A BLAST alignment of the sequences showed similarity with the CHIKV (99%), ZIKV, USUV and WNV genome (100%) (Additional file 1: Figure S1). Using the purified plasmid of each virus, DNA concentration with a known copy number of 4.7 × 10^9^ was prepared. Consequently, 10-fold serial dilutions in water were used to prepare 7 DNA concentrations with copy numbers ranging from 4.7 × 10^8^ to 4.7 × 10^2^ per ml, which were used to prepare the calibration curves for each virus primer and probe. Viral DNA detection was successful for all viruses and the standard curves exhibited linearity over 7 orders of magnitude (Fig. 1). Detection of the highest DNA concentration (4.7 × 10^8^) of CHIKV, ZIKV, USUV and WNV required 10.29, 8.89, 7.19 and 10.78 cycles (Cq), respectively; however, the detection of the lowest DNA concentration (4.7 × 10^2^) required 30.68, 30.32, 30.00 and 31.09 Cq, respectively. The correlation coefficient (*R*^2^ value) was 1.000, 0.998, 0.992 and 1.000 for CHIKV, ZIKV, USUV and WNV, respectively (Fig. 1).

### Specificity of viral primers and probes

The results of the specificity of the primers and probes for ZIKV, USUV, WNV and CHIKV are shown in Table 3. The results indicate that the four viruses were detected using their corresponding primers. The assays were specific for the single target virus; no fluorogenic signal was detected for other tested mosquito-borne viruses. No virus was detected in the *Ae. albopictus-*negative samples. No cross-reaction between these four viruses were detected indicating the high specificity of the assay (Table 3).

**Table 3:** Evaluation of primers and probes specificity

Primer	Cq values				
	ZIKV	USUV	WNV	CHIKV	Negative
ZIKV	20.91	–	–	–	–
USUV	–	22.09	–	–	–
WNV	–	–	17.27	–	–
CHIKV	–	–	–	21.11	–

*Abbreviations*: +, Cq < 36; –, Cq ≥ 36 or no signal

### Quantification of the viral copy number in mosquito heads and bodies

**Fig. 2:**
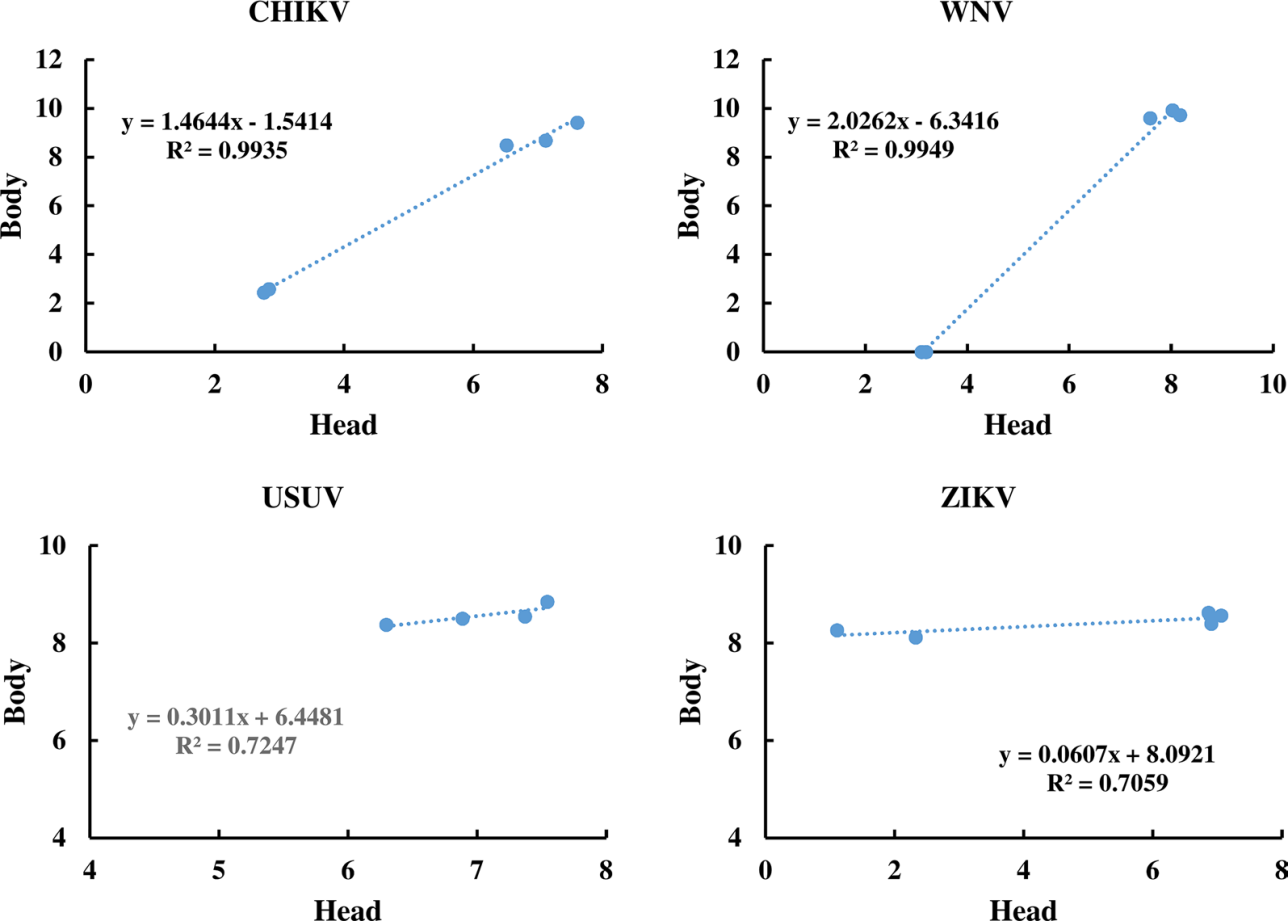
Correlation between the log_10_ virus titer in the heads and bodies of infected mosquitoes

To ensure that the virus infected mosquito bodies were infected, and had relatively homogenous virus copy numbers before using it to spike mosquito pools, the virus copy number was quantified in 5 randomly selected individual bodies with their corresponding heads for each virus (Additional file 2: Table S1). These individuals were randomly selected from the group that showed high virus infection in their heads. The results indicated that the virus was detected in both the head and body and the quantity of the virus in the body and in the corresponding head was positively correlated (Fig. 2). The high regression coefficient (*R*^2^) of 0.994 and 0.995 for CHIKV and WNV, respectively indicated that the assay is highly reproducible. However, *R*^2^ values were rather low for USUV (0.725) and ZIKV (0.710), most probably due to the small size of the tested individuals (*n* = 5). No head with low USUV copy number was detected. Mosquito bodies of the corresponding head with low CHIKV and WNV copy number (~10^3^) exhibited low virus titers (0–10^2^) indicating homogenous virus distribution between the body and the head of infected mosquito. However, interestingly, mosquito bodies corresponding to heads with low ZIKV copy number (10^1^–10^2^) exhibited high virus copy number (~10^8^) indicated less abundance of the virus in the head compared to the body (Fig. 2).

**Fig. 3:**
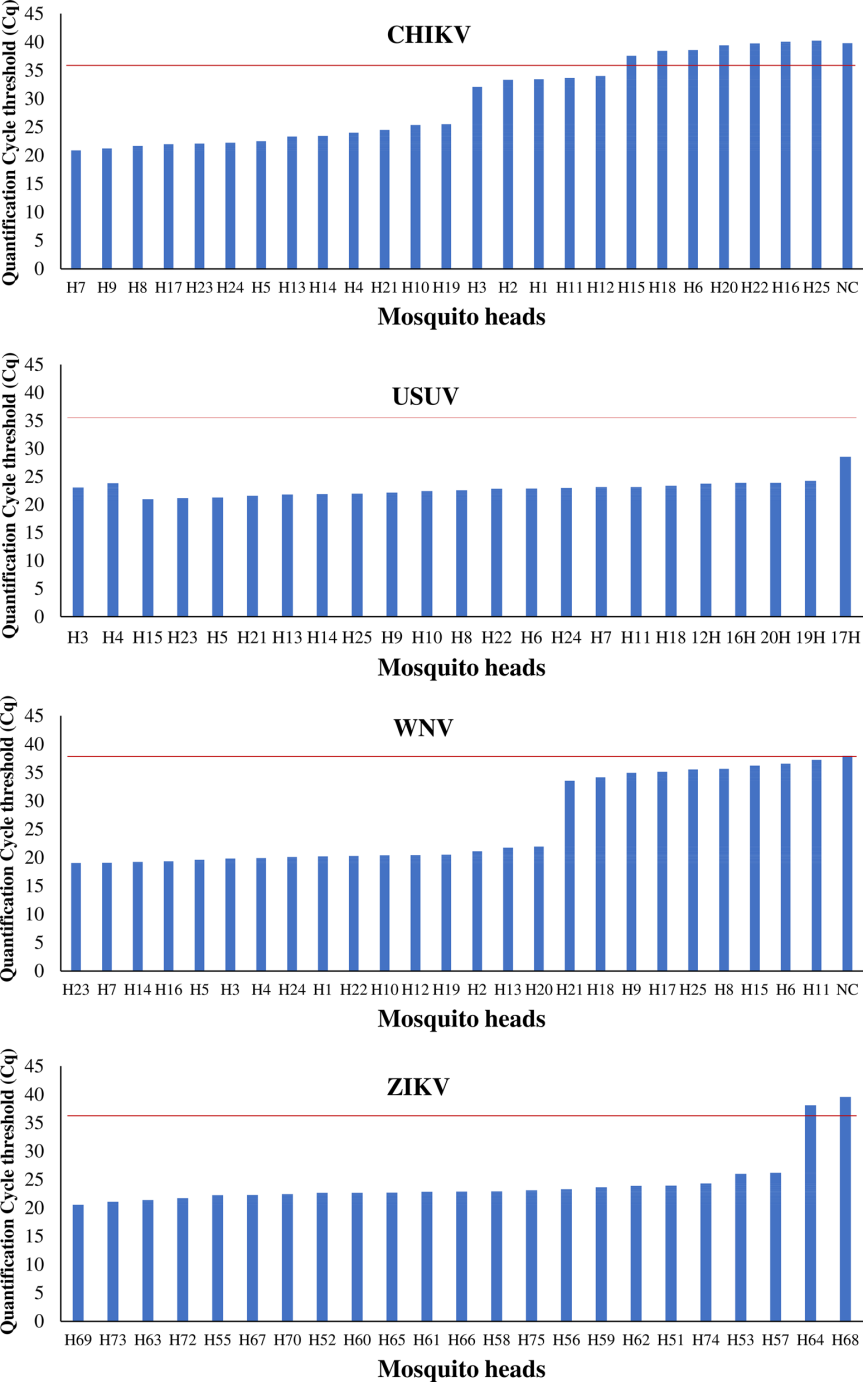
Screening of mosquito-borne virus in the head of infected mosquitoes. The horizontal red bar indicates the cut-off of Cq value 36. Negative control (NC) of USUV and ZIKV did not show a Cq value

The virus infection prevalence was evaluated in all virus inoculated mosquito heads **(**Fig. 3, Additional file 3: Table S2 and the results indicate that 72.5–100% of the head of the inoculated mosquitoes were positive for the virus infection. Based on the Cq value cut-off of 36, heads of all mosquito individuals inoculated with USUV showed virus infection with high virus titer (10^7^–10^8^ virus copy number per head) with a Cq range of 23.07–23.90. The virus infection prevalence in the head of the individual mosquitoes inoculated with CHIKV, WNV and ZIKV were 72, 88 and 91.30%, respectively. For these viruses, some heads showed a virus infection with high virus titer (10^6^–10^8^ per head) with a Cq range of 20.51–26.16 for ZIKV, 19.01–21.93 for WNV and 20.88–25.51 for CHIKV. The heads of some mosquitoes testing positive for CHIKV (20%) and WNV (24%) exhibited low virus titers (10^3^–10^4^ copy numbers per head) with Cq values ranging from 33.55 to 35.65 for WNV and from 32.07 to 33.98 for CHIKV. Bodies corresponding to these heads were not used to spike the uninfected mosquito pools (Additional file 2: Table S1). Based on the positive correlation between the virus copy number in the head and the body of infected mosquitoes, the virus copy number was evaluated in the head of all virus infected mosquitoes (Fig. 2, Additional file 3: Table S2) and subsequently the virus copy number in the corresponding body was calculated as shown in Additional file 4: Table S3. Bodies testing positive with predicted high virus titers were used in the virus detection limit experiment.

### Determination of viral detection limit

**Fig. 4:**
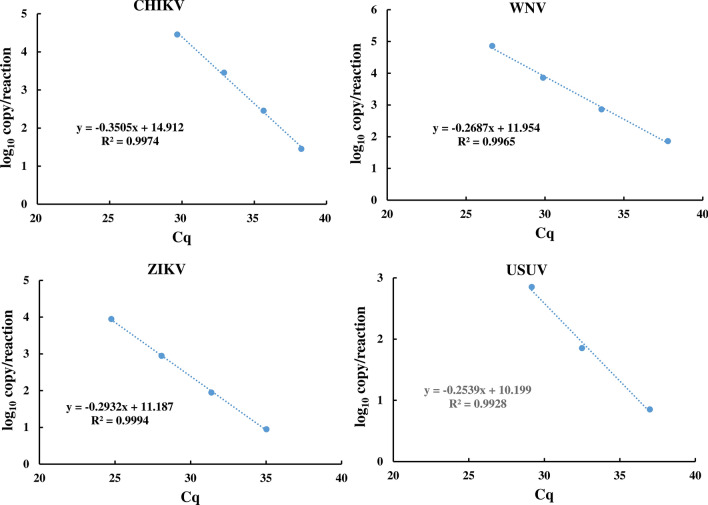
Correlation between the log_10_ copy number per reaction and the quantification cycle threshold (Cq) to determine the mosquito-borne virus detection limit based on the cut-off value of the Cq of 36

The initial detection of MBVs in small pools of mosquitoes (< 100 mosquitoes) containing one virus infected mosquito body indicated the possibility to detect the target viruses (data not shown). Therefore, attempts were made to detect the virus in larger pools of 165, 320 and 1600 uninfected mosquitoes that contained one infected mosquito body. All tested viruses could be detected in all tested pools. For the largest pools of mosquitoes used (1600 mosquitoes spiked with one infected mosquito body), the results indicate the ability to detect CHIKV, WNV, ZIKV and USUV not only by using 10 ng total RNA as a template but also with lower concentrations i.e. 1 and 0.1 ng (Additional file 5: Figure S2). Using the correlation between the virus copy number detected and the different quantities of total RNA, a formula was derived that was used to evaluate the detection limit for each virus (Fig. 4). The detection limits per reaction were 197, 191, 4 and 11 virus copy numbers for CHIKV, WNV, ZIKV and USUV, respectively (Fig. 4, Additional file 6: Table S4).

## Discussion

As demonstrated with the successful management of several plant pests, the SIT has shown great potential for the area-wide management of mosquito populations and hence, the diseases these vectors transmit. The technique has many advantages as it is an environment-friendly and species-specific control method; however, it requires several prerequisites for its implementation [28, 29]. One of these prerequisites is the need to establish a mass-rearing colony of the targeted mosquito species. The mother colony prior to up-scaling is often established from field collected samples which might be infected with MBVs. Taking into account that some MBVs can maintain infections in mosquitoes for up to seven generations within a laboratory colony through vertical and horizontal transmission [30–36], this represents a serious concern for insectary staff handling the mosquito colonies and for the public living in target release sites should sterile female mosquitoes be released accidentally. To avoid such risks, initiating colonies from virus-free material collected from the field is a prerequisite and regular screening of mosquito males and females is recommended to detect any infection in the colony even if infections rates are very low.

Although there are many different detection methods to detect MBVs, the use of RT-qPCR and cell culture were considered the most sensitive techniques [37]. However, the cell culture technique is laborious and time consuming and due to regulations in most countries, requires a Biosafety Level 3 (BSL3) laboratory. The RT-qPCR can be done in a BSL2 laboratory, where the mosquito samples can be homogenized in a virus deactivation solution, i.e. TRIzol or lysis buffer. Both techniques have the advantages of specificity, and sensitivity for the detection of viral RNA; however, cell culture can only detect viable virus particles that can initiate infection and cause cytopathogenic effects (CPE), or viruses have specific antibodies that can be used to detect them using fluorescent focus-forming units (FFU) in the selected cell culture. Viruses that cannot infect these cell cultures or did not induce visible CPE or do not have specific antibodies FFU detection will not be detected unless other techniques are used to confirm the presence of the virus such as RT-qPCR and electron microscopy. RT-qPCR can detect not only viral RNA from viable virus particles but any viral RNA, i.e. mRNA that can be found in the mosquito samples and can be detected with the selected primers and probe sequences. This limitation can be reduced by using multiplex PCR where several sets of primers and probes can be used although this procedure reduces the sensitivity by one log as compared to the single primer set methods [38]. In our study, due to the lack of a BSL3 laboratory, and the time efficiency of the RT-qPCR, this technique was used to detect MBVs.

The detection of MBVs in mosquito pools has been previously studied and the impact of the size of mosquito pools on detection of the virus is well documented [21, 39]. Considering the low virus prevalence in wild mosquitoes, the use of the minimum infection rate (MIR) method was recommended to evaluate mosquito infection rates. In addition, it was shown that increasing the probability to detect MBVs will depend on the size of the mosquito pools [40, 41]. In this study, the infection rate in a mosquito mass-rearing colony initiated from virus-free material is expected to be lower than the infection rate in wild populations. Therefore, larger mosquito pools (320 and 1600 mosquito) were used. Taking into consideration the formula of Gu & Novak [40], the probability of detecting MBVs remains almost the same (0.634 ± 0.011) for the different pools used in this study, even though the mosquito infection rate was significantly different. The infection rate (following the spiking rate of one positive mosquito per pool) was 0.00625, 0.00313 and 0.00067 for the mosquito pools with 160, 320 and 1600 individuals, respectively. This indicates that a larger mosquito pool size compensates for a reduced infection rate and hence, maintains the probability of virus detection. This was confirmed by the detection of the MBVs in the largest mosquito pools (1600) used in this study. These results also agree with the prediction of Gu & Novak [40], who showed that the detection of low levels of mosquito infections requires large samples (i.e. greater than 1600 mosquitoes for obtaining a higher probability of infection (0.8)). It is important to note that the large mosquito pools (1600 mosquitoes) are to be used mainly for virus screening in mosquito mass-rearing facilities where the virus infection titer might be absent, or very low.

Our data not only confirm the possibility of detecting MBVs in larger pools of uninfected mosquitoes (which were larger than the pool sizes tested in previous studies) [21, 37, 40], but they also indicate the theoretical possibility of detecting CHIKV, WNV, ZIKV and USUV in even larger pools of 5.08 × 10^5^, 5.24 × 10^5^, 2.33 × 10^7^ and 8.74 × 10^6^ mosquitoes, respectively, given that one infected mosquito with a high virus titer of 10^8^ copy number is present. These results agree with the results of Jupp et al. [39], who reported the detection of the Rift Valley fever phlebovirus by RT-qPCR in a pool of 16,000 mosquitoes. The large size of screenable mosquito pools predicted in our study might be due to the improvement of the virus detection capacity, i.e. the optimization of RT-qPCR master mix, primers and probe quantity, or due to the difference in the sensitivity of the primers and the probe. Also, this might be due to the assumption of the presence of one infected mosquito with a high copy number which is rarely found in natural mosquito populations unless there is a virus outbreak, where some infected mosquitoes can be found with 10^3.9^ to 10^6.8^ virus copy numbers [15]. Therefore, the sensitivity of the one step RT-qPCR in mosquito pools spiked with one infected mosquito with different virus titers remains to be determined. It is also worth noting that the predicted size of the mosquito pools remains theoretical since using such large numbers of mosquitoes is not practical for the mosquito homogenization and RNA extraction process. In addition, the use of a small number (*n* = 5) of individuals for each virus to assess the correlation between the virus copy number in the head and the corresponding body represents a weakness point in this study that might require further support.

Our data represent an important step to facilitate the implementation of periodic quality control processes to ensure the absence of MBVs in mosquito mass-rearing facilities. The possibility of detecting MVBs in large sample pools will not only reduce the time and manpower needed to conduct this process by at least 50% compared to the efforts required to detect MVBs in small pools, but also provides strong confidence in the negative screening results in mosquito mass-rearing where MBV titers are expected to be absent or extremely low. Although there are advantages of detecting MBVs in large mosquito pools as reported in this study, it has some challenges; for example, the extraction of total RNA from large pools of mosquitoes at once is difficult, as grinding the mosquitoes requires using a large mortar and also requires speed to avoid RNA degradation. Also, the RNA extraction process can only be performed using Trizol methods and none of the RNA extraction kits (that normally facilitate the RNA extraction process) can be used due to the large number of mosquitoes which block the filter used in the RNA extraction kits.

## Conclusions

Based on the overall data presented in this study, it is recommended that field-collected mosquitoes should be kept in a quarantine area and mosquito pools of up to 100 mosquitoes either from collected adults or emerged from collected eggs should be screened to detect any MBVs before initiating a colony in the insectary. In case the screening results turn out to be negative, up-scaling and expanding of the offspring of these mother colonies can be justified. Once a larger mass-rearing colony is established, pools of 1600 mosquitoes can be used to carry out routine screens as part of quality control and bio-safety measures to confirm the absence of MBVs, and to assure the insectary staff and the public that both laboratory colonies and released mosquitoes cannot be associated with any risk of spreading MBVs in the environment during the implementation of SIT programmes for mosquito control.

## Supplementary information

**Additional file 1: Figure S1.** Alignment of CHIKV, USUV, ZIKV and WNV sequence alignment. Viral genome from the database (first lane) was aligned with the archived sequence of the cloned plasmid (second line). *Abbreviations*: F, forward primer; P, probe; R, reverse primer.

**Additional file 2: Table S1.** Determination of the virus copy number in the head of some virus infected mosquitoes. The virus copy number of 6 heads was determined using RT-qPCR and a correlation between the log_10_ virus copy number and the quantification cycle threshold was determined.

**Additional file 3: Table S2.** Determination of the virus copy number in the head of all virus infected mosquitoes. The virus copy number of all heads was determined using a correlation between the log_10_ virus copy number and the quantification cycle threshold (Cq).

**Additional file 4: Table S3.** Determination the viral copy number in infected mosquito bodies used to spike mosquito pools.

**Additional file 5: Figure S2.** The amplification curve of different RNA quantities (10 ng, 1 ng and 0.1 ng per reaction) used as RNA template for RT-qPCR of large mosquito pool (1600 mosquitoes). Head: 10 ng of the head corresponding to the body used to spike the mosquito pool.

**Additional file 6: Table S4.** Determination of the theory detection limit for CKIKV, WNV, USUV and ZIKV using RT-qPCR.

## Data Availability

Data supporting the conclusions of this article are included within the article and its additional files. The datasets used and/or analysed during the present study are available from the corresponding author on reasonable request.

## Abbreviations

R: correlation coefficient
SIT: sterile insect techniques
MBV: mosquito-borne viruses
RT-qPCR: one-step reverse transcriptase quantitative polymerase chain reaction
ITNs: insecticide-treated nets
IRS: indoor residual spraying
RAMPH: rapid analyte measurement platform
CPE: cytopathogenic effects
FFU: fluorescent focus-forming units
MIR: minimum infection rate
