# Supplementary figures and images for: High sensitivity of one-step real-time reverse transcription quantitative PCR to detect low virus titers in large mosquito pools
Source: Parasit Vectors. 2020 Sep 9;13:460. doi: 10.1186/s13071-020-04327-4 (PMC7488135; doi:10.1186/s13071-020-04327-4)

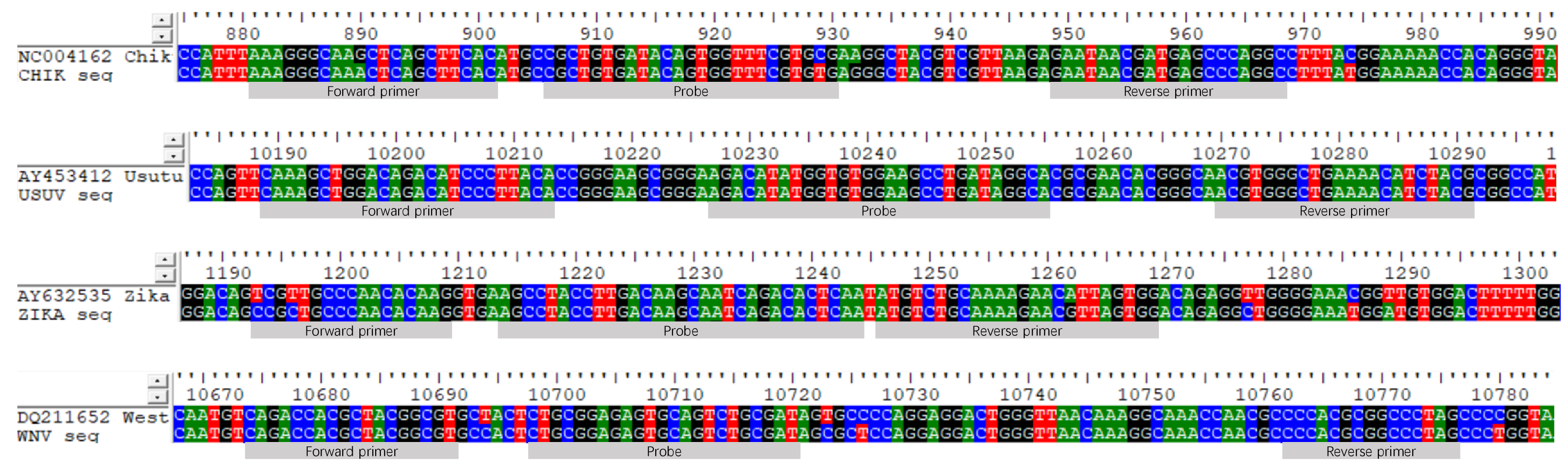

Supplement: Supplementary file 1 — Additional file 1: Figure S1. Alignment of CHIKV, USUV, ZIKV and WNV sequence alignment. Viral genome from the database (first lane) was aligned with the archived sequence of the cloned plasmid (second line). Abbreviations: F, forward primer; P, probe; R, reverse primer. [file 13071_2020_4327_MOESM1_ESM.tif]

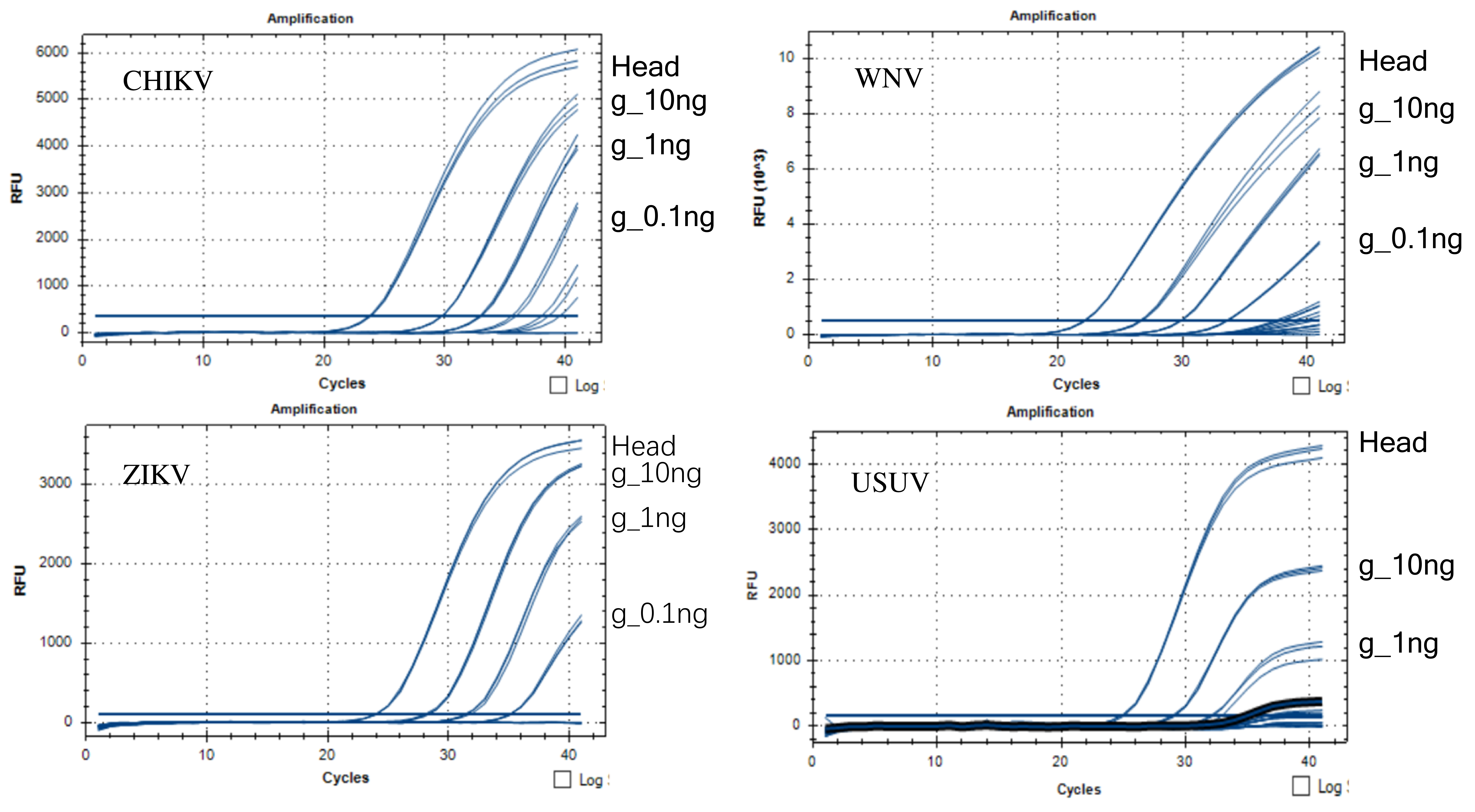

Supplement: Supplementary file 5 — Additional file 5: Figure S2. The amplification curve of different RNA quantities (10 ng, 1 ng and 0.1 ng per reaction) used as RNA template for RT-qPCR of large mosquito pool (1600 mosquitoes). Head: 10 ng of the head corresponding to the body used to spike the mosquito pool. [file 13071_2020_4327_MOESM5_ESM.tif]
