# Supplementary material for: High sensitivity of one-step real-time reverse transcription quantitative PCR to detect low virus titers in large mosquito pools
Source: Parasit Vectors. 2020 Sep 9;13:460. doi: 10.1186/s13071-020-04327-4 (PMC7488135; doi:10.1186/s13071-020-04327-4)
Supplement: Supplementary file 4 — Additional file 4: Table S3. Determination the viral copy number in infected mosquito bodies used to spike mosquito pools. [file 13071_2020_4327_MOESM4_ESM.docx]

**Additional file 4: Table S3**. Determination the viral copy number in infected mosquito bodies used to spike mosquito pools

| Samples | Head Cq | Measured | | Predicted |
| --- | --- | --- | --- | --- |
|  |  | Head Log_10__Total copy number | Body Log_10__Total copy number | Body Log_10__Total copy number^e^ |
| CHIKV 24^a^ | 22.21 | 7.79 | 9.47 | 9.87 |
| CHIKV 5^b^ | 22.47 | 7.78 | 8.84 | 9.85 |
| CHIKV 4^b^ | 23.99 | 7.49 | 8.95 | 9.43 |
| CHIKV 23^c^ | 22.06 | 7.96 | 9.37 | 10.12 |
| CHIKV 13^c^ | 23.3 | 7.54 | 9.44 | 9.5 |
| CHIKV 10^d^ | 25.34 | 7.12 | 8.68 |  |
| CHIKV 19^d^ | 25.51 | 6.52 | 8.48 |  |
| CHIKV 14^d^ | 23.44 | 7.61 | 9.41 |  |
| CHIKV 16^d^ | 40.04 | 2.76 | 2.43 |  |
| CHIKV 25^d^ | 40.24 | 2.84 | 2.58 |  |
| USUV 8^a^ | 22.55 | 7.23 | 8.67 | 8.63 |
| USUV 22^b^ | 22.82 | 6.87 | 8.85 | 8.52 |
| USUV 11^b^ | 23.14 | 7.04 | 8.55 | 8.57 |
| USUV 24^c^ | 22.99 | 7.13 | 8.53 | 8.59 |
| USUV 6^c^ | 22.85 | 6.89 | 8.73 | 8.52 |
| USUV 3^d^ | 23.07 | 7.54 | 8.84 |  |
| USUV 4^d^ | 23.82 | 7.37 | 8.54 |  |
| USUV 19^d^ | 24.25 | 6.89 | 8.50 |  |
| USUV 17^d^ | 28.52 | 6.29 | 8.37 |  |
| WNV 24^a^ | 22.20 | 8.52 | 9.88 | 10.89 |
| WNV 1^b^ | 22.17 | 8.39 | 10.03 | 10.64 |
| WNV 2^b^ | 23.26 | 8.14 | 9.89 | 10.13 |
| WNV 22^c^ | 22.16 | 8.33 | 10.10 | 10.50 |
| WNV 10^c^ | 22.37 | 8.56 | 9.89 | 10.97 |
| WNV 12^d^ | 20.49 | 8.17 | 9.72 |  |
| WNV 13^d^ | 21.87 | 7.59 | 9.60 |  |
| WNV 20^d^ | 21.77 | 8.03 | 9.91 |  |
| WNV 6^d^ | 37.71 | 3.19 | 0.00 |  |
| WNV 11^d^ | 37.90 | 3.10 | 0.00 |  |
| ZIKV 75^a^ | 23.97 | 7.73 | 9.38 | 8.56 |
| ZIKV 59^b^ | 24.94 | 7.16 | 10.03 | 8.53 |
| ZIKV 58^b^ | 24.86 | 7.38 | 9.14 | 8.54 |
| ZIKV 56^c^ | 24.81 | 7.41 | 9.11 | 8.54 |
| ZIKV 66^c^ | 23.86 | 7.25 | 9.27 | 8.53 |
| ZIKV 74^d^ | 24.02 | 6.90 | 8.39 |  |
| ZIKV 53^d^ | 25.70 | 7.06 | 8.56 |  |
| ZIKV 57^d^ | 25.90 | 6.86 | 8.62 |  |
| ZIKV 71^d^ | 39.30 | 2.32 | 8.11 |  |
| ZIKV 54^d^ | 37.78 | 1.11 | 8.25 |  |

^a^used to spike 100 mg pool

^b^used to spike 200 mg pool

^c^used to spike 1000 mg pool

^d^used to determine the virus copy number in both the head and the body

^e^predicted based on the copy number in the corresponding head
